# Profiling of Volatile Organic Compounds in Wild Indigenous Medicinal Ginger (*Zingiber barbatum* Wall.) from Myanmar

**DOI:** 10.3390/metabo10060248

**Published:** 2020-06-15

**Authors:** Musavvara Kh. Shukurova, Yonathan Asikin, Yanhang Chen, Miyako Kusano, Kazuo N. Watanabe

**Affiliations:** 1 Graduate School of Life and Environmental Sciences, University of Tsukuba, Ibaraki 305-8572, Japan; yanhangchen@outlook.com; 2 Department of Bioscience and Biotechnology, Faculty of Agriculture, University of the Ryukyus, Okinawa 903-0213, Japan; y-asikin@agr.u-ryukyu.ac.jp; 3 Faculty of Life and Environmental Science, University of Tsukuba, Ibaraki 305-8572, Japan; kusano.miyako.fp@u.tsukuba.ac.jp (M.K.); watanabe.kazuo.fa@u.tsukuba.ac.jp (K.N.W.); 4 Tsukuba-Plant Innovation Research Center, University of Tsukuba, Ibaraki 305-8572, Japan; 5 RIKEN Center for Sustainable Resource Science, Kanagawa 230-0045, Japan

**Keywords:** *Zingiber barbatum*, *Zingiber*, volatile organic compounds (VOCs), chemical markers, solid-phase microextraction (SPME), gas chromatography time-of-flight-mass spectrometry (GC-TOF-MS)

## Abstract

The emissions of volatile organic compounds (VOCs) strongly depend on the plant species and are differently represented in specific taxa. VOCs have a degree of chemical diversity and also can serve as chemotaxonomic markers. *Zingiber barbatum* Wall. is a wild medicinal ginger plant endemic to Myanmar whose VOC composition has never been screened before. In this study, we screened the rhizome of *Z. barbatum* to identify the VOC composition by the application of gas chromatography combined with time-of-flight-mass spectrometry (GC-TOF-MS). The resulting VOC profile of *Z. barbatum* showed that it consists mainly of monoterpenes (21%) and sesquiterpenes (30%). Intraspecific similarities and dissimilarities were found to exist between *Z. barbatum* genotypes in terms of VOC composition. Four accessions (ZO191, ZO223, ZO217, and the control accession ZO105) collected from the Shan State and Mandalay region of Myanmar were found to share a similar VOC profile, while two accessions (ZO64 and ZO160) collected from the Bago region were found to vary in their VOC profiles compared with the control accession. The two identified compounds, i.e., α-bergamotene and β-(*E*)-guaiene may serve as discriminative chemical markers for the characterization of *Z. barbatum* species collected in these three geographical regions of Myanmar. This study represents a first attempt to identify and describe the VOCs in the medicinal species *Z. barbatum* that have not been reported to date.

## 1. Introduction

Volatile organic compounds (VOCs) are involved in various vital activities of plants. To communicate and interact, plants produce, store, and emit VOCs, which manifests as quick signaling between distant organs to enhance resistance to an upcoming stress [1,2,3]. Different plant organs produce different groups of VOCs [4], and their emissions strongly depend on the plant species [5]. Separate plant lineages often resolve the same problem, such as attracting pollinators, enhancing resistance to stress, or interacting with other plants, by adopting different chemical solutions [6]. As secondary metabolites, VOCs have a high degree of chemical diversity and are differently represented in specific taxa; thus, they can also serve as chemotaxonomic markers [3,6]. Based on their biosynthetic origin and chemical structure, the major classes of plant VOCs are volatile terpenes (such as monoterpenes, sesquiterpenes, diterpenes, and isoprenes) and oxygenates (such as aldehydes, alcohols, ketones, and esters), phenylpropanoids, derivatives of fatty acids and amino acids, and moderately volatiles compounds, such as furanocoumarins and their derivatives [3,7,8].

Wild medicinal species, while underutilized, are valued as a source of useful bioactive compounds possessing medicinal properties. The genus *Zingiber*, of the Zingiberaceae family, comprises 144 species, most of which are considered to be medicinal [9]. *Zingiber* species are commonly used as a spice, a food, and a dietary supplement and have been used as a traditional remedy in Asian and Southeast Asian countries for a long time. The phytochemical composition of *Zingiber officinale* (an edible ginger), *Zingiber montanum* (a cassumunar ginger), *Zingiber zerumbet* (a bitter ginger), and *Zingiber mioga* (a Japanese ginger) is quite well reported. The rhizome is recognized to be a valuable part of the plant due to its possession of several biological activities, including anti-inflammatory [10,11,12], antioxidant [13,14], antimicrobial [15,16,17,18] and anticancer [19,20,21,22] activity. The unique aroma, flavor, and bioactive properties of *Zingiber* species are related to the combination of their chemical constituents: volatile compounds (constituting the essential oil) and phenolic compounds (gingerols, shogaols, and paradols) [23,24]. The essential oil of *Zingiber* species is a complex mixture of VOCs that consists mainly of terpenoids with different functional groups that vary in structure [23,24,25]. The mono- and sesquiterpenes are the major classes of VOCs characteristic of the species in the genus *Zingiber* [23,24]. The species-specific and “unique” volatile substances reported for *Z. officinale* are α-zingiberene, geranial, and ar-curcumene; those reported for *Z. montanum* are sabinene, (*Z*)-ocimene, and terpinen-4-ol; those reported for *Z. zerumbet* are zerumbone and pinene; and those reported for *Z. mioga* are β-phellandrene and β-elemene [18,25,26,27,28,29]. These wide variety of compounds play a major role in the bioactivity of *Zingiber* species, for instance, α-zingiberene, camphene, α-farnesene, and β-sesquiphellandrene are attributed to the antioxidant activity of *Z. officinale* [30]. Zerumbone is the main constituent in *Z. zerumbet* and reported to induce apoptosis in pancreatic carcinoma cells and possess the anti-inflammatory and chemopreventive potential [20,21,30]. Sabinene is the major constituents of *Z. cassumunar* and possesses strong anti-inflammatory and antimicrobial activity [27,31]. From the taxonomic or systematic point of view, the distinctive feature of the plants is not the production of an essential oil; rather, it is the biosynthetically specific group of substances (e.g., flavonoids, sulfur compounds, and terpenoids) characteristic to specific species, since the more that substances can be derived from the biosynthetic pathway, the more specific this group can be for certain taxa [32].

*Zingiber barbatum* Wall. is a wild-type ginger plant, belongs to the genus *Zingiber*, of the family Zingiberaceae. *Z. barbatum* is an underexploited medicinal species, commonly known as “Meik-thalin” or “Pwe-au” in Myanmar [33]. Myanmar has a strong cultural heritage and a unique traditional medicine system, that plays an essential role in daily health care [34]. Due to its anti-inflammatory and analgesic properties, the rhizome of *Z. barbatum* is used in ethnomedicine in Myanmar to treat gout and relieve joint, bone, and muscle pains [35]. *Z. barbatum* has a long history of use as a herbal remedy; however, very little is known about this species. Studies on *Z. barbatum*’s genetics based on morphological and molecular markers revealed a high degree of genetic variability among *Z. barbatum* genotypes [36]. The composition of both the volatile and non-volatile constituents of *Z. barbatum* has not been screened to date; furthermore, the pharmacological properties related to its bioactivity remain untapped.

Hydrodistillation and solvent extraction are the methods most commonly used to collect VOCs from plant matrices [37,38,39]. However, these methods have several disadvantages, including a low recovery rate, destruction of sample’s matrix, and the use of destructive organic solvents [39]. Headspace solid-phase microextraction (HS-SPME) coupled with gas chromatography (GC) is reported to be a non-destructive, efficient, and solvent-free method for collecting VOCs from plants [4,40].

The main objective of this study was to identify and characterize the VOC composition in *Z. barbatum* species. To identify *Z. barbatum*’s VOC composition by headspace (HS) sampling of emitted VOCs using solid-phase microextraction (SPME) methods [40], we used the rhizome as the experimental material. The collected HS samples were directly analyzed by using gas chromatography combined with time-of-flight-mass spectrometry (GC-TOF-MS). We also aimed to reveal some “unique” species-specific compounds, that may serve as chemotaxonomic markers in genetic diversity studies.

## 2. Results

### 2.1. The VOC Profile in the Headspace of Six Z. barbatum Rhizomes

The rhizome of six *Z. barbatum* accessions from the collection of the Gene Research Center of the University of Tsukuba in Japan (GRC UT), which are originating from Myanmar was screened for provisional identification of VOCs in HS of samples using the SPME method.

**Figure 1:**
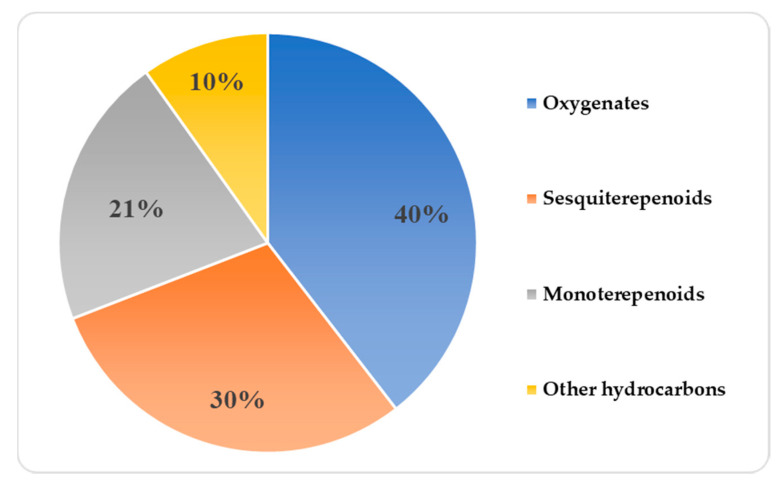
A chart showing the proportions of identified classes of organic compounds of the 81 putatively annotated peaks in the headspace (HS) samples of *Zingiber barbatum* species. The “oxygenates” pie chart consists of a combination of the alcohols, aldehydes, ketones, and esters classes.

We obtained VOC profile data on 24 samples (two biological × two analytical replicates) and extracted 362 mass spectral peaks as a data matrix. The detected peaks were provisionally identified using an automated annotation pipeline, following Kusano et al. [40]. The mass spectra (MS) and retention index (RI) of each peak were matched against the reference reported in the libraries [41,42,43,44] used to identify VOCs. Of the 362 peaks, 81 detected peaks were considered to be putatively annotated compounds as their mass spectra showed a match value > 800 and the RIs of the corresponding peaks were 20 units less than those reported in the corresponding libraries. The molecular formula of each annotated peak, the class of each chemical compound, and the CAS registered number were identified using chemistry databases PubChem [45] and ChemSpider [46]. The molecular formula of each annotated peak allowed us to determine the class and calculate the proportion of the main organic compounds. The proportion of chemical compounds by class in the profile of *Z. barbatum* samples was calculated by counting the number of annotated compounds in each class as a total of 100%, and then calculating the proportion in each class relative to the summary value (Figure 1). The result showed that the main classes of organic compounds in the HS samples of *Z. barbatum* species were monoterpenoids and sesquiterpenoids, followed by oxygenates (i.e., alcohols, aldehydes, ketones, and esters) and other hydrocarbons. As shown in Figure 1, while the group of “oxygenates” occupies a larger proportion in the pie chart (40%), each class separately comprises a small fraction of the group (of the total 100%) and remains lower compared with the proportions of monoterpenes and sesquiterpenes.

### 2.2. Multivariate Data Analysis (MVDA) of Omics Data

#### 2.2.1. Principal Component Analysis (PCA) and Orthogonal Partial Least Square Projection to Latent Structures Differential Analysis (OPLS-DA)

There are multiple available statistical approaches to the analysis, modeling, and validation of data used in metabolomics [47,48]. Multivariate data analyses (MVDAs), such as principal component analysis (PCA) and orthogonal partial least squares projection to latent structures differential analysis (OPLS-DA), can be used to draw conclusions about and to obtain meaning from metabolomics datasets [49].

PCA and OPLS-DA were performed using the SIMCA software (version 14.0, Umetrics AB, Umeå, Sweden) to visualize the differences between classes in the complex VOC datasets. First, PCA was performed to identify the metabolic differences between the examined *Z. barbatum* samples. The result showed the absence of a clear separation between samples (*n* = 24), indicating that the PCA model was not suitable for the discriminative analysis of the data obtained in this study (Supplementary Figure S1).

**Figure 2:**
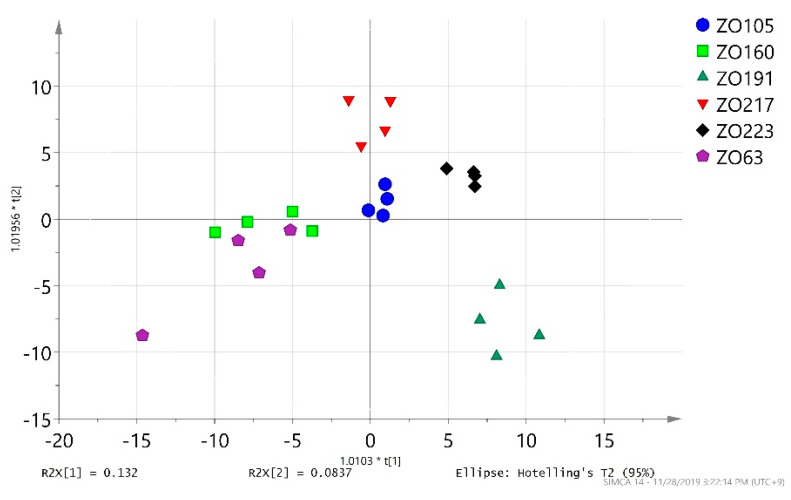
The scatter plot of the orthogonal partial least squares projection to latent structures differential analysis (OPLS-DA) scores generated from the gas chromatography combined with time-of-flight-mass spectrometry (GC-TOF-MS) data (*n* = 24) on *Z. barbatum* accessions. The first two principle components of the principal component analysis (PCA) accounted for a total of 22% of the variance.

In contrast, the scatter plot of the OPLS-DA generated from the GC-TOF-MS data (*n* = 24) clearly separated the assessed groups [50,51]. The OPLS-DA gathered the group of ZO105 samples in the center of the scatter plot of scores; therefore, the ZO105 sample group was chosen as a provisional control sample group for subsequent data analyses (Figure 2). The first two principle components of the PCA accounted 13% (R2X[1] = 0.132) and 8% (R2X[2] = 0.0837) of the variance. A permutation plot with 500 permutations for the OPLS-DA model was created to determine the validity and degree of overfitting. The Y-intercept (Q^2^Y) on the permutation graph is a measure that checks for overfitting (Supplementary Figure S2). The model attained a Q^2^Y value of −0.493 in the permutation plot. However, the difference between Q^2^ (Q2(cum) = 0.235) and R^2^Y (R2Y (cum) = 0.8) was 0.5 (from an admissible 0.3), indicating that the model slightly overfitted the data [52].

#### 2.2.2. Principal Components Analysis (PCA) and Hierarchical Cluster Analysis (HCA)

**Figure 3:**
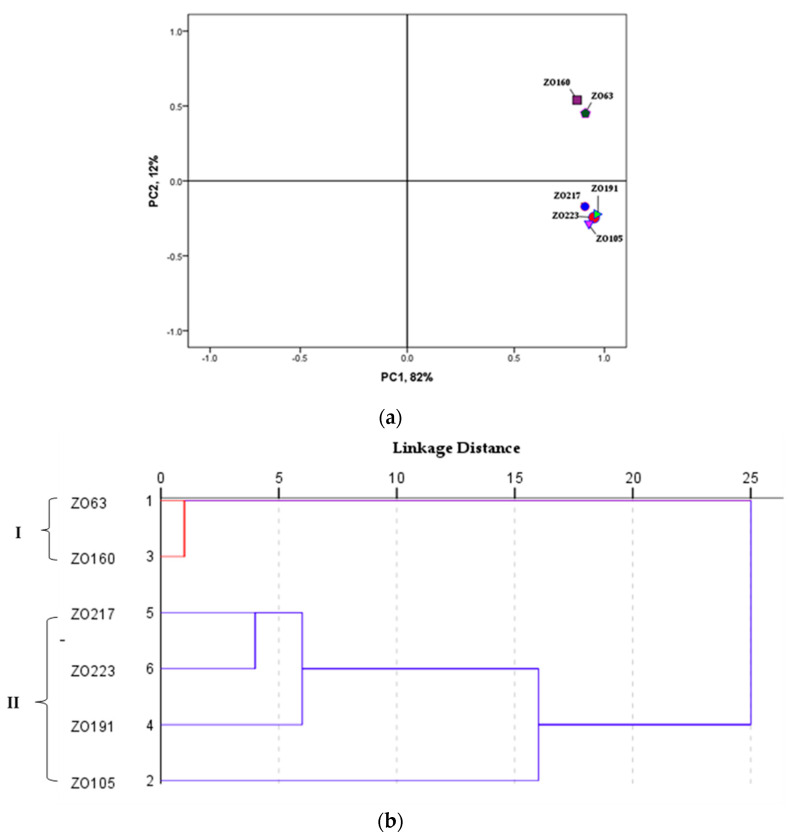
(**a**) The generated PCA plot and (**b**) hierarchical cluster analysis (HCA) dendrogram showing the clustering pattern based on the Euclidean distance between *Z. barbatum* groups. The principal components PC1 and PC2 accounted for 94% of the total variance.

We included a total of 81 volatile organic compounds in the PCA and hierarchical cluster analysis (HCA), which were based on the Euclidean distance from the average value (two biological × two analytical replicates) of the data after normalization using SPSS software version 24 (IBM Corp, Armonk, NY, USA) (Figure 3).

The PCA analysis showed a clear separation between groups and formed two groups (Group I and II, Figure 3a) that were situated in the positive quadrants of the generated PCA plot. The contribution of the first two components PC1 and PC2 to the variance was 82% and 12%, respectively, with a total cumulative contribution to the variance of 94%. The accessions ZO63 and ZO160 from the Bago region were grouped together in Group I, whereas Group II was comprised of accessions ZO105, ZO191, ZO217, and ZO223 from the Mandalay region and Shan state.

The HCA dendrogram indicated a similarity between clusters and a hierarchical relationship and generated a solution with two clusters. The number of clusters (Clusters I and II, Figure 3b) was determined by using the rescaled distances in the dendrogram based on a cut-off point where the distances among combinations of clusters increase substantially as the between-group variability increases in terms of volatile composition. These clusters are formed in the same groups in the generated PCA plot: the vertical axis correlated positively with Cluster I and horizontal axis correlated positively with Cluster II (Figure 3a).

### 2.3. Composition of VOCs in the VOC Profile of Z. barbatum Samples

Changes in the VOC composition in each *Z. barbatum* accession compared with the control (ZO105) were recorded by subtracting the average of the normalized responses of the annotated peaks (as a log_2_-transformed value) and assessing the extent of any significant difference (with a false discovery rate (FDR) < 0.05). The transformed log_2_ and FDR values allowed us to present the data as the fold-change value of a metabolite concentration normalized relative to the control. The results show that two accessions ZO191 and ZO223 have a similar VOC profile to the control accession ZO105. The levels of the annotated VOCs between these accessions did not show any significant difference (FDR < 0.05) (Supplementary Table S1). The VOC profile of accession ZO217 was similar to that of the control accession ZO105, except for two compounds: terepinen-4-ol and 2,5-bornanediol (Supplementary Table S1). Two accessions ZO63 and ZO160 significantly (FDR < 0.05) differed from the control accession ZO105. In particular, 11 compounds and 14 compounds in the VOC profile of ZO63 and ZO160, respectively, were different when compared with the compounds in the VOC profile of the control accession ZO105 (Supplementary Table S2). The monoterpenoid identified to be discriminative was elemol acetate, and the sesquiterpenoids identified to be discriminative were β-farnesene, α-ylangene, α-zingiberene, germacrene A, β-bisabolene, β-sesquiphellandrene, valencene, cuparene, and selina-5,11-diene (Supplementary Table S2). Moreover, two compounds α-bergamotene and β-(*E*)-guaiene were not detected in the VOC profile of the control accession ZO105.

A Tukey’s test based on the normalized values revealed that, of the 81 compounds, 24 had significant differences in content between the six examined *Z. barbatum* species (Table 1). The compounds that showed a significant difference between accessions were butyl pivalate, α-phellandrene, α-terpinene, β-phellandrene, 3-methyldecane, γ-terpinene, (4*E*)-7-methyl-4-decene, terpinolene, (*E*)-3-caren-2-ol, octyl acetate, terpinen-4-ol, (*Z*)-sabinene hydrate acetate, bornyl acetate, valeric acid, 2,7,10-trimethyldodecane, 2,5-bornanediol, α-ylangene, 12-chloro-5-dodecyne, β-farnesene, α-zingiberene, (*E*)-β-guaiene, β-bisabolene, β-sesquiphellandrene, 7-epi-α-Selinene, and elemol acetate.

## 3. Discussion

We applied a non-targeted method for the identification of VOCs in six *Z. barbatum* species in order to detect qualitative differences between the analyzed samples and to identify/classify them through the degree of similarity of their fingerprints to those reported in available libraries. The rhizome of six *Z. barbatum* species was screened to produce a profile of the total VOC composition. As was reported for the other species in the *Zingiber* genus [23,25,26,28], the result showed that the major class of VOCs identified in the VOC profile of *Z. barbatum* was terpenoids. Twenty-one percent of identified compounds were monoterpenes (C_10_H_16_), and 30% of identified compounds were sesquiterpenes (C_15_H_24_) (Figure 1). Due to the biological activities that most terpenoids possess, they have found a wide range of applications in pharmacology, medicine, and biotechnology. Compounds belonging to the class of monoterpenes usually have a strong aroma and odor and are commonly used as fragrances in perfumes and cosmetics [53]. Most monoterpenes exhibit biological activity, including antibacterial, anti-inflammatory, and antitumor activity [54,55]. Compounds belonging to the class of sesquiterpene possess antimicrobial and anti-insecticidal activity and have an effect on the regulation and prevention of oxidative damage and inflammation-mediated biological damage [53,56]. A steam-heated medicinal product derived from milled rhizome of *Z. barbatum* is used in ethnomedicine in Myanmar. The ambient temperature, the vapor pressure, and the size of the monoterpene pool in plant tissues are the factors that have the greatest influence on monoterpene emissions. Sesquiterpenes possess high reactivity and a low vapor pressure [4]. In this regard, we suggest that the healing properties of *Z. barbatum* may be due to the bioactivity of the monoterpene class of compounds, which may be activated during the steam-heating process.

The emissions of VOCs depend on the environmental conditions under which a plant grows. We hypothesized that *Z. barbatum* species collected from a diverse eco-geographical region of Myanmar would possess different VOC profiles. The log_2_-transformed data revealed that four *Z. barbatum* accessions ZO105, ZO190, ZO217, and ZO223 collected from Shan state and the Mandalay region have a similar VOC profile (Supplementary Table S1). Two accessions ZO63 and ZO160 collected from the Bago region varied in terms of the VOC composition in their profiles. However, the results of the Tukey’s test revealed significant differences in 24 compounds between the examined samples.

In order to investigate the relationships between *Z. barbatum* genotypes based on the identified VOCs, PCA was performed and a HCA dendrogram was generated. The PCA grouped accessions ZO63 and ZO160, which were collected from the Bago region, into Group I and the accessions ZO105, ZO191, ZO217, and ZO223, which were collected from the Mandalay region and Shan state, into Group II, which is consistent with the log_2_ transformed data (Figure 3a). The generated HCA dendrogram also supports the between-group discrimination and positively correlates with the groups of the PCA plot (Figure 3b). The distances between links show dissimilarities among the VOCs identified in the studied *Z. barbatum* genotypes. The HCA revealed that accessions ZO63 and ZO160 from the Bago region are closely linked to each other (Cluster I) in terms of VOC composition. Interestingly, that accession ZO217 from Shan State and accession ZO223 from the Mandalay region are more closely linked to each other (Cluster II) in terms of VOC composition than to the accessions collected from the same region (ZO191 from Shan State and ZO105 from the Mandalay region), respectively. The characterization of genetic diversity based on morphological and molecular markers reported by other researchers [36] has also revealed a high degree of variation among *Z. barbatum* accessions that enabled them to be divided into two morphotype groups with comparatively higher genetic diversity. The cluster analysis based on morphological characteristics grouped together the above-mentioned accessions ZO63 and ZO160 into one cluster (Cluster 2), while accession ZO105 was included in Cluster 1. The same trend was observed based on a characterization using molecular markers [36]. The results allow us to suggest that chemical diversity exists between *Z. barbatum* accessions and VOCs could be useful as “chemotaxonomic markers” for the characterization of inter-and intraspecific variability among *Z. barbatum* species from different eco-geographical regions of Myanmar.

Most *Zingiber* species are aromatic; however, very limited information is available regarding which compound or group of compounds is responsible for their unique fragrance. Different *Zingiber* species have different VOC compositions, contributing to their unique aroma. *Z. barbatum* has a pleasant camphor−citrus aroma that was found to be very different between genotypes during olfactory testing. Most of the aroma-contributing VOCs are reported to be monoterpenoids [57]. Geraniol and its derivatives geranial, geranyl acetate, geraniol, and citronellol are the major aroma-contributing compounds that have been reported for *Z. officinale* rhizome, which is characterized by a pleasant fresh citrus aroma and was reported to be the most aroma-active compound in ginger [58]. Geraniol diphosphate (GDP) is a universal monoterpenoid precursor to the production of geraniol by geraniol synthase in plants [59]. Even though geraniol-related compounds are structurally similar, they differ in terms of aromatic properties and the composition of compounds and can vary due to the environment in which they are cultivated and the maturity of the rhizome [57]. Seventeen monoterpenoids have been identified in the VOC profile of *Z. barbatum* that might contribute to the light lemon–mint or lemon–camphor aroma in this species.

Plants maintain the memory of any stress event they have experienced. VOCs are able to shape a plant’s stress memory because their volatility allows them to the quickly reach distant plant parts [60]. In this study, all the examined plants were grown in uniformity, i.e., in a pot in the field of GRC UT, at the same altitude and under the same ecological conditions. Consequently, four *Z. barbatum* accessions (ZO190, ZO217, ZO223, and ZO150) collected from different eco-geographical regions of Myanmar possessed similar VOC profiles. However, the variation in the VOC composition observed in ZO63 and ZO160 was probably related to the influence of geographical and ecological (abiotic, biotic) factors on the production of VOCs in plants by reference to the “plant memory” [60]. Sanli and Karadogan [61] reported that high altitudes increased the sesquiterpene constituents in *Kundmannia anatolica* Hub.-Mor. Demasi et al. [62] reported qualitative and quantitative intra-species variations in secondary metabolites in the aroma of *Lavandula angustifolia* Mill. due to the influence of altitude; sesquiterpenes were present in higher amounts in the aroma of the low-altitude populations. Negative correlations between secondary metabolites and latitude and positive correlations between secondary metabolites and temperature were reported by Guo et al. [63] for *Scutellaria baicalensis*. The authors noted that a high temperature is beneficial to the accumulation of most secondary metabolites in the root of *S. baicalensis* [63]. It has been also reported that such VOCs as monoterpenes (camphene and pinene) actively participate in the mechanisms leading to systemic acquired resistance (SAR). Bergamotenes serve as pheromones for some insects; thus, plants defend themselves by attracting the predators of herbivorous pests by producing such natural pheromones. The green leaf volatiles (GLVs), such as *Z*-3-hexenyl acetate, which are rapidly released after mechanical damage occurs to leaf tissues, induce the resistance of wheat plants to the fungal pathogen *Fussarium graminearum* and reduce the damage that occurs to maize plants during cold stress [60,64,65].

Given the aforesaid, we suggest that the observed similarities and differences between VOCs in *Z. barbatum* accessions might be related to plant memory (i.e., their place of origin). The four accessions ZO190, ZO217, ZO223, and ZO105, which were found to have similar VOC profiles, might be more tolerant to abiotic and biotic stresses in a natural environment. The accessions ZO63 and ZO160 might be more sensitive to the stresses in a natural environment, due to which their VOC profiles were different compared with that of the control accession ZO105. VOCs play an important role in the evolutionary process as a response to biotic and abiotic stresses and in plant’s adaptation to its environment [6,66,67]. Further studies with the inclusion of more accessions per population for the identification VOCs in *Z. barbatum* are required to confirm this conclusion.

## 4. Materials and Methods

### 4.1. Plant Materials

#### 4.1.1. Sample Selection

Six accessions of *Z. barbatum* from the collection of the Gene Research Center of the University of Tsukuba (GRC UT) (Tsukuba, Japan) were used in this study. Table A1 contains a list of these accessions together with the appropriate identification code number and information about the plants’ origin and collection site.

The experimental plants were grown during the planting season from May 2019 to October 2019. The rhizome of each individual candidate sample was planted in a plastic pot with dimensions of 30 × 50 cm and placed in the open field at GRC UT.

*Z. barbatum* rhizomes were obtained during a field study on the exploration of plant genetic resources as part of a Myanmar−Japan cooperative project. Rhizomes were transferred to Japan via the Standard Material Transfer Agreement (SMTA) under the International Treaty on Plant Genetic Resources for Food and Agriculture (ITPGRFA) of the United Nations (UN) Food and Agriculture Organization (FAO). The plant materials were maintained as a living collection in a greenhouse of at GRC UT. The selection of samples was based on the geographical distribution, the availability of plant materials (rhizomes), and the detected genetic diversity as reported in a previous study [36].

#### 4.1.2. Experimental Design, Sample Collection, and Sample Preparation

The experiment was carried out using two biological replicates for each accession. Two analytical replications were used for each biological replicate.

Rhizome bit samples (≈25 g per sample) were collected and washed thoroughly under running tap water to remove soil residues and traces of other impurities. The samples were then dried using paper towel for 15 min.

The rhizome samples were cut into 0.5 cm^2^ pieces using disposable stainless-steel surgical blades (No. 24, Kai Industries Co., Ltd., Gifu, Japan) and changed individually for each sample. Approximately 10 g of chopped sample was transferred to homogenization tube. The samples were immediately frozen in liquid nitrogen and stored at −30 °C to quench metabolism.

### 4.2. Sample Processing

#### 4.2.1. Homogenization and Aliquot Preparation

Samples were cryohomogenized to a fine powder in MB2000 Multi-bead Shocker (Yasui Kikai Co., Ltd., Osaka, Japan) in two rounds at 2800 rpm for 16 s per cycle.

Each frozen powder sample was weighed (10 mg) and then dissolved in sterilized distilled water. A stock solution of an aliquot with a 10 mg/mL concentration was diluted to the final working concentration of 500 μg/mL and stored at −30 °C until use.

#### 4.2.2. Chemicals

The chemicals and reagents used in the study were all of analytical grade. The EPA524.2 fortification solution (surrogate standard mixture) was purchased from Sigma-Aldrich Japan (Tokyo, Japan). The *n*-alkane standard solution for determination of the RI (C8–C20) was purchased from Fluka Chemical (Tokyo, Japan). The other chemicals were purchased from Nacalai Tesque (Kyoto, Japan) or Wako Pure Chemical Industries (Osaka, Japan).

#### 4.2.3. VOC Extraction and Headspace Collection by a SPME Fiber

The VOCs were extracted by following the procedure described by Kusano et al. [32] with some modifications. The collection of all VOCs from the HS samples was carried out using a preconditioned solid-phase microextraction (SPME) fiber with dimensions of 50/30 μm DVB/CAR/PDMS (Supelco, St. Louis, MO, USA).

The analytes for VOC extraction were prepared in 20 mL headspace vials (Supelco, St. Louis, MO, USA) consisting of 1.0 mL of sample solution (500 μg/mL), 1.0 mL of 100 mM EDTA (pH 7.5), and 10 μL of EPA 524.2 fortification solution (20 μg/mL of fluorobenzene, 4-bromofluorobenzene, and 1,2-dichloro-benzene-d4) in methanol, which was used as an internal standard (IS). Samples were incubated for 10 min at 80 °C, and then the VOCs were extracted over a period of 20 min. Samples were introduced randomly through a CTC PAL autosampler (CTC Analytics AG, Zwingen, Switzerland).

### 4.3. GC-TOF-MS Analysis

The samples were injected into an Agilent 6890N (Agilent Technologies, Wilmington, DE, USA) gas chromatograph equipped with a Rxi-5Sil MS column (30 m × 0.25 mmID × 0.25 μm; RESTEK, Bellefonte, PA, USA). We used the splitless injection mode and helium as a carrier gas at a constant flow rate of 1 mL/min. The GC temperature program was as follows: the initial column temperature 55 °C was maintained for 3 min, then increased to 150 °C at the rate of 15 °C/min, then increased to 200 °C at the rate of 3 °C/min, and finally maintained at 200 °C for 2 min. The back-inlet temperature was kept at 250 °C. Mass spectral analysis was performed on a Pegasus III 4D TOF-MS (LECO, St. Joseph, MI, USA). The MS ionization energy (voltage) was set at 70 eV. The ionization source temperature was 200 °C; the MS scan range (*m*/*z*) was 29–500 amu; and the acquisition rate was 30 spectra/s.

### 4.4. Data Processing and Provisional Identification of VOCs in HS Samples of Z. barbatum Species

The data processing was performed by following the workflow scheme described in Kusano et al. (2016) with some modifications.

The data obtained from the GC-TOF-MS analysis were transformed into the NetCDF format using Leco ChromaTOF version 4.71.0.0 (LECO, St. Joseph, MI, USA). All data-preprocessing procedures, including baseline correction, peak alignment, smoothing, time-window setting, and deconvolution by the hierarchical multi-curve resolution (H-MCR) method (Jonsson et al., 2006) were carried out using MATLAB 7.0 (Mathworks, Natick, MA, USA). The normalization of peaks was done by calculating the area of the mass spectral values for the internal standards using MATLAB R2011b (Mathworks). Then, the peaks in the data matrix were normalized by the cross-contribution compensating multiple standard normalization (CCMN) method [40], which allows for the elimination of systematic variations and subsequent analysis. The adjusted mass spectra that were obtained by applying the H-MCR method were matched against reference mass spectra from different libraries using the NIST mass spectral search program (version 2.2) and the custom software for peak annotation developed by Kusano et al. [40]. The commercially available libraries used for the identification and estimation of VOCs were the Adams Library (4th edition) [41], the Terpenoids Library [42], the VocBinBase Library [43], NIST14 [44], and the Wiley’s FFNSC Library (Mass Spectra of Flavors and Fragrances of Natural and Synthetic Compounds, 3rd edition).

The similarity (≥850 or 900) and the RI difference (<|30 units|) were used to identify the same or very similar compounds from the referenced libraries and NIST05 [40]. When the standard deviation (SD) of the absolute RI difference between these compounds was less than 8.8 units, we applied a similarity of ≥800 with differences less than 20 units to determine whether peaks were from a putatively annotated compound.

### 4.5. Statistical Analysis

The multivariate analysis was done using SIMCA 14.0 software (Umetrics AB, Umeå, Sweden) and IBM’s SPSS software version 24.0 (IBM Corp, Armonk, NY, USA). The VOC profile data were log_2_–transformed, and then statistically analyzed using the LIMMA package [68]. FDR correction for multiple testing [69] was performed in the R environment for statistical computing (version 3.5.0).

## 5. Conclusions

The HS-SPME approach was applied for non-targeted GC-TOF-MS profiling of the VOC composition in *Z. barbatum* species collected from three different eco-geographical regions of Myanmar. This approach was shown to be suitable for profiling the VOC composition in *Z. barbatum* species A total of 21% of the identified VOCs were found to be monoterpenoids, and a total of 30% of the identified VOCs were found to be sesquiterpenoids. Intraspecies variation in VOC composition was observed in the accessions collected from the Bago region (ZO63 and ZO160) and the accessions collected from the Mandalay region (ZO105 and ZO223) and Shan State (ZO190 and ZO217). Based on the identified VOCs, we revealed a clear separation and a close relationship between the six *Z. barbatum* species collected from three different regions of Myanmar. The chemical diversity that exists among these *Z. barbatum* species may be useful to chemotaxonomic and diversity studies. Two of the identified volatile compounds α-bergamotene and β-(*E*)-guaiene might serve as discriminative chemical markers in the assessment of *Z. barbatum* germplasms, since they were identified only in the profiles of accessions ZO63 and ZO160. Taken together, the results of the present study represent the first report of the VOCs in the underexploited medicinal species *Z. barbatum* from Myanmar.

## Figures and Tables

**Table 1:** Significant differences between identified volatile organic compounds (VOCs) in six *Z. barbatum* species.

Compounds	ZO63	ZO105	ZO160	ZO191	ZO217	ZO223
1,3,5-Cycloheptatriene	0.00 ± 0.00 ^a^	0.00 ± 0.00 ^a^	0.00 ± 0.00 ^a^	0.00 ± 0.00 ^a^	0.00 ± 0.00 ^a^	0.00 ± 0.00 ^a^
Hexanal	0.00 ± 0.00 ^a^	0.01 ± 0.00 ^a^	0.01 ± 0.00 ^a^	0.01 ± 0.00 ^a^	0.00 ± 0.00 ^a^	0.00 ± 0.00 ^a^
2-methylbutan-2-yl acetate	0.00 ± 0.00 ^a^	0.00 ± 0.00 ^a^	0.00 ± 0.00 ^a^	0.00 ± 0.00 ^a^	0.00 ± 0.00 ^a^	0.00 ± 0.00 ^a^
(*Z*)-3-Octene	0.01 ± 0.00 ^a^	0.01 ± 0.00 ^a^	0.01 ± 0.00 ^a^	0.00 ± 0.00 ^a^	0.00 ± 0.00 ^a^	0.00 ± 0.00 ^a^
Heptanal	0.00 ± 0.00 ^a^	0.00 ± 0.00 ^a^	0.00 ± 0.00 ^a^	0.00 ± 0.00 ^a^	0.00 ± 0.00 ^a^	0.00 ± 0.00 ^a^
γ-Butyrolactone	0.00 ± 0.00 ^a^	0.00 ± 0.00 ^a^	0.00 ± 0.00 ^a^	0.00 ± 0.00 ^a^	0.00 ± 0.00 ^a^	0.00 ± 0.00 ^a^
α-Thujene	0.30 ± 0.09 ^a^	0.56 ± 0.34 ^a^	0.22 ± 0.07 ^a^	0.46 ± 0.28 ^a^	0.15 ± 0.14 ^a^	0.16 ± 0.09 ^a^
2-Octanone	0.01 ± 0.00 ^a^	0.01 ± 0.00 ^a^	0.00 ± 0.00 ^a^	0.00 ± 0.00 ^a^	0.00 ± 0.00 ^a^	0.00 ± 0.00 ^a^
Sabinene	0.25 ± 0.13 ^a^	0.32 ± 0.15 ^a^	0.25 ± 0.28 ^a^	0.53 ± 0.32 ^a^	0.10 ± 0.04 ^a^	0.16 ± 0.16 ^a^
Butyl pivalate	0.08 ± 0.04 ^a,b^	0.06 ± 0.03 ^a,b,c^	0.06 ± 0.03 ^a,b,c^	0.02 ± 0.01 ^b,c^	0.09 ± 0.03 ^a^	0.01 ± 0.00 ^c^
Decane	0.08 ± 0.01 ^a^	0.08 ± 0.01 ^a^	0.08 ± 0.01 ^a^	0.09 ± 0.01 ^a^	0.08 ± 0.01 ^a^	0.08 ± 0.01 ^a^
Octanal	0.01 ± 0.00 ^a^	0.01 ± 0.00 ^a^	0.01 ± 0.01 ^a^	0.01 ± 0.00 ^a^	0.01 ± 0.01 ^a^	0.01 ± 0.00 ^a^
α-Phellandrene	0.12 ± 0.02 ^a,b^	0.21 ± 0.13 ^a^	0.09 ± 0.01 ^a,b^	0.12 ± 0.06 ^a,b^	0.04 ± 0.01 ^b^	0.07 ± 0.02 ^b^
1,4-Dichlorobenzene	0.04 ± 0.00 ^a^	0.04 ± 0.01 ^a^	0.04 ± 0.01 ^a^	0.05 ± 0.00 ^a^	0.04 ± 0.01 ^a^	0.04 ± 0.00 ^a^
α-Terpinene	0.33 ± 0.08 ^a,b^	0.64 ± 0.43 ^a^	0.21 ± 0.06 ^a,b^	0.36 ± 0.19 ^a,b^	0.09 ± 0.05 ^b^	0.17 ± 0.08 ^a,b^
o-Cymene	0.76 ± 0.29 ^a^	1.51 ± 0.90 ^a^	0.65 ± 0.14 ^a^	0.79 ± 0.49 ^a^	0.52 ± 0.04 ^a^	0.59 ± 0.15 ^a^
Limonene	0.02 ± 0.01 ^a^	0.03 ± 0.02 ^a^	0.01 ± 0.00 ^a^	0.02 ± 0.01 ^a^	0.01 ± 0.00 ^a^	0.01 ± 0.01 ^a^
β-Phellandrene	0.12 ± 0.03 ^a,b^	0.23 ± 0.13 ^a^	0.09 ± 0.02 ^b^	0.14 ± 0.07 ^a,b^	0.03 ± 0.03 ^b^	0.07 ± 0.03 ^b^
3-Methyldecane	0.00 ± 0.00 ^a^	0.00 ± 0.00 ^a^	0.00 ± 0.00 ^a,b^	0.00 ± 0.00 ^b^	0.00 ± 0.00 ^a^	0.00 ± 0.00 ^b^
2-Methyldecane	0.02 ± 0.01 ^a^	0.02 ± 0.00 ^a^	0.03 ± 0.01 ^a^	0.03 ± 0.01 ^a^	0.02 ± 0.00 ^a^	0.03 ± 0.01 ^a^
γ-Terpinene	0.48 ± 0.12 ^a,b^	0.94 ± 0.68 ^a^	0.30 ± 0.10 ^a,b^	0.55 ± 0.30 ^a,b^	0.13 ± 0.07 ^b^	0.23 ± 0.12 ^a,b^
1-Octanol	0.01 ± 0.00 ^a^	0.01 ± 0.00 ^a^	0.01 ± 0.00 ^a^	0.01 ± 0.00 ^a^	0.01 ± 0.01 ^a^	0.01 ± 0.00 ^a^
(*Z*)-Sabinenhydrate	0.02 ± 0.02 ^a^	0.01 ± 0.01 ^a^	0.01 ± 0.01 ^a^	0.02 ± 0.01 ^a^	0.00 ± 0.01 ^a^	0.01 ± 0.01 ^a^
(*E*)-Sabinenhydrate	0.07 ± 0.07 ^a^	0.04 ± 0.05 ^a^	0.03 ± 0.04 ^a^	0.08 ± 0.05 ^a^	0.02 ± 0.02 ^a^	0.03 ± 0.03 ^a^
(4*E*)-7-Methyl-4-decene	0.02 ± 0.00 ^a,b^	0.01 ± 0.00 ^a,b^	0.01 ± 0.00 ^a,b^	0.01 ± 0.00 ^a^	0.01 ± 0.00 ^b^	0.01 ± 0.00 ^b^
4-Tolualdehyde	0.00 ± 0.00 ^a^	0.00 ± 0.00 ^a^	0.00 ± 0.00 ^a^	0.00 ± 0.00 ^a^	0.00 ± 0.00 ^a^	0.00 ± 0.00 ^a^
Terpinolene	0.11 ± 0.03 ^a,b^	0.21 ± 0.15 ^a^	0.07 ± 0.02 ^a,b^	0.12 ± 0.07 ^a,b^	0.03 ± 0.02 ^b^	0.05 ± 0.03 ^a,b^
p-Cymenene	0.00 ± 0.00 ^a^	0.01 ± 0.01 ^a^	0.00 ± 0.00 ^a^	0.00 ± 0.00 ^a^	0.00 ± 0.00 ^a^	0.00 ± 0.00 ^a^
Decane	0.05 ± 0.01 ^a^	0.06 ± 0.01 ^a^	0.05 ± 0.01 ^a^	0.07 ± 0.01 ^a^	0.05 ± 0.00 ^a^	0.06 ± 0.02 ^a^
Nonanal	0.03 ± 0.03 ^a^	0.06 ± 0.05 ^a^	0.04 ± 0.02 ^a^	0.04 ± 0.05 ^a^	0.05 ± 0.02 ^a^	0.03 ± 0.02 ^a^
2,4,6-Trimethyldecane	0.01 ± 0.00 ^a^	0.01 ± 0.00 ^a^	0.01 ± 0.00 ^a^	0.01 ± 0.00 ^a^	0.01 ± 0.00 ^a^	0.01 ± 0.01 ^a^
(*E*)-3-caren-2-ol	0.00 ± 0.00 ^a,b^	0.001±0.00 ^a^	0.00 ± 0.00 ^ab^	0.00 ± 0.00 ^a,b^	0.00 ± 0.00 ^b^	0.00 ± 0.00 ^a,b^
Octyl acetate	0.01 ± 0.00 ^a^	0.01 ± 0.00 ^a,b^	0.01 ± 0.00 ^a,b^	0.01 ± 0.00 ^a,b^	0.01 ± 0.00 ^a,b^	0.01 ± 0.00 ^b^
L-camphor	0.00 ± 0.00 ^a^	0.00 ± 0.00 ^a^	0.01 ± 0.00 ^a^	0.01 ± 0.00 ^a^	0.01 ± 0.00 ^a^	0.01 ± 0.00 ^a^
1-Nonanol	0.00 ± 0.00 ^a^	0.00 ± 0.00 ^a^	0.00 ± 0.00 ^a^	0.00 ± 0.00 ^a^	0.00 ± 0.00 ^a^	0.00 ± 0.00 ^a^
Tetrahydrolinalyl acetate	0.00 ± 0.00 ^a^	0.00 ± 0.00 ^a^	0.00 ± 0.00 ^a^	0.00 ± 0.00 ^a^	0.00 ± 0.00 ^a^	0.00 ± 0.00 ^a^
Terpinen-4-ol	0.72 ± 0.04 ^b^	1.25 ± 0.21 ^a^	0.63 ± 0.10 ^a,c^	0.79 ± 0.09 ^b^	0.26 ± 0.06 ^c^	0.87 ± 0.32 ^b^
Naphthalene	0.00 ± 0.00 ^a^	0.00 ± 0.00 ^a^	0.00 ± 0.00 ^a^	0.00 ± 0.00 ^a^	0.00 ± 0.00 ^a^	0.00 ± 0.00 ^a^
5,6-Dimethylundecane	0.02 ± 0.00 ^a^	0.01 ± 0.00 ^a^	0.02 ± 0.00 ^a^	0.02 ± 0.00 ^a^	0.02 ± 0.00 ^a^	0.02 ± 0.00 ^a^
(*Z*)-Sabinene hydrate acetate	0.01 ± 0.00 ^a,b^	0.03 ± 0.01 ^a^	0.01 ± 0.00 ^b^	0.02 ± 0.01 ^a,b^	0.01 ± 0.00 ^b^	0.01 ± 0.01 ^b^
1,3-Di-tert-butylbenzene	0.03 ± 0.01 ^a^	0.03 ± 0.01 ^a^	0.03 ± 0.01 ^a^	0.03 ± 0.01 ^a^	0.03 ± 0.02 ^a^	0.02 ± 0.01 ^a^
4,6-Dimethyldodecane	0.00 ± 0.00 ^a^	0.00 ± 0.00 ^a^	0.00 ± 0.00 ^a^	0.00 ± 0.00 ^a^	0.00 ± 0.00 ^a^	0.00 ± 0.00 ^a^
Bornyl acetate	0.15 ± 0.04 ^a^	0.05 ± 0.02 ^b^	0.12 ± 0.03 ^a^	0.03 ± 0.01 ^b^	0.03 ± 0.01 ^b^	0.02 ± 0.01 ^b^
Terpinen-4-ol acetate	0.02 ± 0.01 ^a^	0.03 ± 0.01 ^a^	0.01 ± 0.01 ^a^	0.03 ± 0.01 ^a^	0.01 ± 0.00 ^a^	0.02 ± 0.01 ^a^
Tridecane	0.00 ± 0.00 ^a^	0.00 ± 0.00 ^a^	0.00 ± 0.00 ^a^	0.00 ± 0.00 ^a^	0.00 ± 0.00 ^a^	0.00 ± 0.00 ^a^
Valeric acid	0.01 ± 0.00 ^a^	0.00 ± 0.00 ^b^	0.00 ± 0.00 ^a,b^	0.00 ± 0.00 ^b^	0.00 ± 0.00 ^b^	0.00 ± 0.00 ^b^
2,7,10-Trimethyldodecane	0.00 ±0.00 ^a^	0.00 ± 0.00 ^a^	0.00 ± 0.00 ^a^	0.01 ± 0.00 ^a^	0.00 ± 0.00 ^a^	0.00 ± 0.00 ^a^
2,5-Bornanediol	0.00 ± 0.00 ^b^	0.00 ± 0.00 ^a^	0.00 ± 0.00 ^b^	0.00 ± 0.00 ^a^	0.00 ± 0.00 ^b^	0.00 ± 0.00 ^a^
β-Terpinyl acetate	0.09 ± 0.02 ^a^	0.10 ± 0.04 ^a^	0.07 ± 0.04 ^a,b^	0.07 ± 0.02 ^a,b^	0.03 ± 0.01 ^b^	0.04 ± 0.02 ^a,b^
3-Hydroxy-2,4,4-trimethylpentyl 2-methylpropanoate	0.03 ± 0.00 ^a^	0.03 ± 0.00 ^a^	0.03 ± 0.00 ^a^	0.03 ± 0.00 ^a^	0.03 ± 0.00 ^a^	0.03 ± 0.01 ^a^
α-Ylangene	0.01 ± 0.01 ^a^	0.00 ± 0.00 ^b^	0.01 ± 0.00 ^a,b^	0.00 ± 0.00 ^b^	0.00 ± 0.00 ^b^	0.00 ± 0.00 ^b^
β-Elemene	0.00 ± 0.00 ^a^	0.00 ± 0.00 ^a^	0.01 ± 0.00 ^a^	0.00 ± 0.00 ^a^	0.00 ± 0.00 ^a^	0.00 ± 0.00 ^a^
Tetradecane	0.04 ± 0.01 ^a^	0.03 ± 0.01 ^a^	0.03 ± 0.01 ^a^	0.04 ± 0.01 ^a^	0.04 ± 0.00 ^a^	0.03 ± 0.01 ^a^
γ-Elemene	0.01 ± 0.02 ^a^	0.00 ± 0.00 ^a^	0.01 ± 0.01 ^a^	0.00 ± 0.00 ^a^	0.00 ± 0.00 ^a^	0.00 ± 0.00 ^a^
α-Bergamotene	0.00 ± 0.00 ^a^	0.00 ±0.00 ^a^	0.00 ± 0.00 ^a^	0.00 ± 0.00 ^a^	0.00 ± 0.00 ^a^	0.00 ± 0.00 ^a^
12-Chloro-5-dodecyne	0.01 ± 0.00 ^a^	0.00 ± 0.00 ^b^	0.00 ± 0.00 ^a,b^	0.00 ± 0.00 ^a,b^	0.00 ± 0.00 ^b^	0.00 ± 0.00 ^b^
Dihydrocurcumene	0.01 ± 0.01 ^a^	0.00 ± 0.00 ^a^	0.01 ± 0.00 ^a^	0.00 ± 0.00 ^a^	0.00 ± 0.00 ^a^	0.00 ± 0.00 ^a^
β-Farnesene	0.03 ± 0.01 ^a^	0.00 ± 0.00 ^b^	0.02 ± 0.01 ^a^	0.00 ± 0.00 ^b^	0.00 ± 0.00 ^b^	0.00 ± 0.00 ^b^
Selina-5,11-diene	0.02 ± 0.02 ^a^	0.00 ± 0.00 ^a^	0.01 ± 0.01 ^a^	0.00 ± 0.00 ^a^	0.00 ± 0.00 ^a^	0.00 ± 0.00 ^a^
3,11-Acoradiene	0.02 ± 0.02 ^a^	0.01 ± 0.00 ^a^	0.02 ± 0.02 ^a^	0.00 ± 0.00 ^a^	0.00 ± 0.00 ^a^	0.00 ± 0.00 ^a^
α-Curcumene	0.00 ± 0.00 ^a^	0.00 ± 0.00 ^a^	0.00 ± 0.00 ^a^	0.00 ± 0.00 ^a^	0.00 ± 0.00 ^a^	0.00 ± 0.00 ^a^
γ-Curcumene	0.04 ± 0.04 ^a^	0.00 ± 0.00 ^a^	0.03 ± 0.02 ^a^	0.00 ± 0.00 ^a^	0.00 ± 0.00 ^a^	0.00 ± 0.00 ^a^
Cuparene	0.22 ± 0.20 ^a^	0.02 ± 0.01 ^a^	0.19 ± 0.11 ^a^	0.03 ± 0.02 ^a^	0.013 ± 0.01 ^a^	0.10 ± 0.01 ^a^
γ-Amorphene	0.00 ± 0.00 ^a^	0.00 ± 0.00 ^a^	0.00 ± 0.00 ^a^	0.00 ± 0.00 ^a^	0.00 ± 0.00 ^a^	0.00 ± 0.00 ^a^
Germacrene A	0.44 ± 0.43 ^a^	0.00 ± 0.00 ^a^	0.39 ± 0.22 ^a^	0.00 ± 0.00 ^a^	0.00 ± 0.00 ^a^	0.00 ± 0.00 ^a^
α-Zingiberene	0.38 ± 0.34 ^a^	0.02 ± 0.02 ^b^	0.34 ± 0.17 ^a,b^	0.02 ± 0.02 ^b^	0.01 ± 0.01 ^b^	0.01 ± 0.00 ^b^
Valencene	0.05 ± 0.05 ^a^	0.00 ± 0.00 ^a^	0.05 ± 0.03 ^a^	0.00 ± 0.00 ^a^	0.00 ± 0.00 ^a^	0.00 ± 0.00 ^a^
2,4-Di-tert-butylphenol	0.78 ± 0.08 ^a^	0.70 ± 0.14 ^a^	0.80 ± 0.10 ^a^	0.80 ± 0.11 ^a^	0.81 ± 0.13 ^a^	0.66 ± 0.19 ^a^
(*E*)-β-Guaiene	0.14 ± 0.11 ^a^	0.00 ± 0.00 ^b^	0.11 ± 0.06 ^a,b^	0.00 ± 0.00 ^b^	0.00 ± 0.00 ^b^	0.00 ± 0.00 ^b^
β-Bisabolene	0.05 ± 0.04 ^a^	0.00 ± 0.00 ^b,c^	0.05 ± 0.02 ^a,b^	0.01 ± 0.00 ^b,c^	0.00 ± 0.00 ^c^	0.00 ± 0.00 ^b,c^
σ-Cadinene	0.00 ± 0.00 ^a^	0.00 ± 0.00 ^a^	0.00 ± 0.00 ^a^	0.00 ± 0.00 ^a^	0.00 ± 0.00 ^a^	0.00 ± 0.00 ^a^
β-Sesquiphellandrene	1.19 ± 0.80 ^a^	0.11 ± 0.07 ^b^	1.32 ± 0.68 ^a^	0.17 ± 0.11 ^b^	0.09 ± 0.03 ^b^	0.09 ± 0.05 ^b^
7-epi-α-Selinene	0.08 ± 0.07 ^a^	0.00 ± 0.00 ^b^	0.07 ± 0.03 ^a,b^	0.00 ± 0.00 ^b^	0.00 ± 0.00 ^b^	0.00 ± 0.00 ^b^
1-Iodoundecane	0.00 ± 0.00 ^a^	0.00 ± 0.00 ^a^	0.00 ± 0.00 ^a^	0.00 ± 0.00 ^a^	0.00 ± 0.00 ^a^	0.00 ± 0.00 ^a^
(*E*)-γ-Macrocarpene	0.01 ± 0.01 ^a^	0.00 ± 0.00 ^a^	0.01 ± 0.00 ^a^	0.00 ± 0.00 ^a^	0.00 ± 0.00 ^a^	0.00 ± 0.00 ^a^
Germacrene B	0.01 ± 0.01 ^a^	0.00 ± 0.00 ^a^	0.01 ± 0.01 ^a^	0.00 ± 0.00 ^a^	0.00 ± 0.00 ^a^	0.00 ± 0.00 ^a^
2,2,4-Trimethyl-1,3-pentadienol diisobutyrate	0.02 ± 0.00 ^a^	0.02 ± 0.00 ^a^	0.02 ± 0.01 ^a^	0.02 ± 0.01 ^a^	0.02 ± 0.01 ^a^	0.01 ± 0.00 ^a^
2-Allyl-1,4-dimethoxy-3-methylbenzene	0.02 ± 0.01 ^a^	0.01 ± 0.00 ^a^	0.01 ± 0.01 ^a^	0.03 ± 0.02 ^a^	0.03 ± 0.02 ^a^	0.02 ± 0.01 ^a^
Hexadecane	0.01 ± 0.00 ^a^	0.01 ± 0.00 ^a^	0.01 ± 0.01 ^a^	0.02 ± 0.01 ^a^	0.01 ± 0.00 ^a^	0.01 ± 0.01 ^a^
Elemol acetate	0.05 ± 0.01 ^a^	0.00 ± 0.00 ^c^	0.02 ± 0.02 ^b^	0.00 ± 0.00 ^c^	0.00 ± 0.00 ^c^	0.00 ± 0.00 ^c^
2-Tetradecyloxirane	0.00 ± 0.00 ^a^	0.00 ± 0.00 ^a^	0.00 ± 0.00 ^a^	0.00 ± 0.00 ^a^	0.00 ± 0.00 ^a^	0.00 ± 0.00 ^a^

Compounds with the same letter are not significantly different (Tukey HCD test, α = 0.05). Each value shows the mean ± standard deviation (std) of four replications (two biological and two analytical replications) for each examined accession of *Z. barbatum*.

## Supplementary Materials

The following are available online at https://www.mdpi.com/2218-1989/10/6/248/s1, Figure S1: Scores plot of PCA of the VOC profile of *Z. barbatum* (*n* = 24). Figure S2: Permutation test plot to validate the OPLS-DA score model (500 permutations). Table S1: Putatively annotated VOCs in the profile of *Z. barbatum* identified by HS-SPME method. Table S2: Putatively annotated VOCs in profile of ZO63 and ZO160 accession significantly differ compare to the control accession ZO105.

## Appendix A

**Table A1:** List of accessions, the appropriate identification code number, the plant origins, and collection site.

Species	Accession (GRC UT)	Status	Collection Site		Altitude (m a.s.l.)	Year of Acquisition by SMTA
			City	Region/State		
*Z. barbatum*	ZO 63	Landrace	Nattalin	Bago	4	2004
*Z. barbatum*	ZO 105	Landrace	Pyon oo lwin	Mandalay	1070	2004
*Z. barbatum*	ZO 160	Landrace	Thayarwaddy	Bago	15	2007
*Z. barbatum*	ZO 191	Landrace	Pin Da Ya	Shan	1164	2008
*Z. barbatum*	ZO 217	Landrace	Aung Ban	Shan	1286	2009
*Z. barbatum*	ZO 223	Landrace	Kyauk Pa Daung	Mandalay	595	2009
